# Autologous culture method improves retention of tumors’ native properties

**DOI:** 10.1038/s41598-020-77238-0

**Published:** 2020-11-24

**Authors:** Yao Tang, Qian Xu, Meiling Yan, Yimin Zhang, Ping Zhu, Xianghong Li, Limin Sang, Ming Zhang, Wenhe Huang, Lianxing Lin, Jundong Wu, Yue Xin, Junhui Fu, Li Zhang, Shuming Zhang, Jiang Gu

**Affiliations:** 1 Provincial Key Laboratory of Infectious Diseases and Molecular Immunopathology, Department of Pathology and Pathophysiology, Shantou University Medical College, Shantou, 515041 Guangdong China; 2 Department of Pathology, Beijing University Cancer Hospital, Beijing, 100142 China; 3 Dalian Municipal Central Hospital, Dalian, 116033 Liaoning China; 4 Cancer Hospital of Shantou University Medical College, Shantou, 515041 Guangdong China; 5 Shantou Central Hospital, Shantou, 515041 Guangdong China; 6 Jinxin Research Institute for Reproductive Medicine and Genetics, Chengdu Jinjiang Hospital for Maternal and Child Health Care, Chengdu, 610066 Sichuan China

**Keywords:** Drug screening, Cancer models, Cancer microenvironment, Cancer models, Cancer therapy, Tumour heterogeneity, Biological techniques, Cancer, Drug discovery, Medical research, Cancer microenvironment, Cancer models, Cancer therapeutic resistance, Oncology, Chemotherapy

## Abstract

No current in vitro tumor model replicates a tumor’s in vivo microenvironment. A culturing technique that better preserves a tumor’s pathophysiological conditions is needed for some important clinical applications, including personalized drug-sensitivity/resistance assays. In this study, we utilized autologous serum or body fluid to build a 3D scaffold and grow a patient’s tumor. We named this technique “3D-ACM” (autologous culture method). Forty-five clinical samples from biopsies, surgically removed tumor tissues and malignant body fluids were cultured with 3D-ACM. Traditional 3D-FBS (fetal bovine serum) cultures were performed side-by-side for comparison. The results were that cells cultured in 3D-ACM rebuilt tissue-like structures, and retained their immuno-phenotypes and cytokine productions. In contrast, the 3D-FBS method promoted mesenchymal cell proliferation. In preliminary chemo drug-sensitivity assays, significantly higher mortality was always associated with FBS-cultured cells. Accordingly, 3D-ACM appears to more reliably preserve a tumor’s biological characteristics, which might improve the accuracy of drug-testing for personalized cancer treatment.

## Introduction

Cancer patients are highly individualized in their responses to anti-cancer regimens, so it is important to tailor a cancer treatment to a particular patient. Each tumor has its own physiological and biological characteristics; to retain these properties in vitro, an individualized tumor culture is necessary. No tumor grows in two-dimensional (2D) form in a host, so three-dimensional (3D) culture techniques are being vigorously pursued to improve cancer research and provide higher accuracy in drug discovery^1^. However, current 3D in vitro cancer models poorly replicate the native environment of a patient’s tumor, often resulting in the use of drugs that perform well in these models but fail in clinical trials^2,3^. One of the significant weaknesses is that most cultures are performed on single cell populations (cloned cells or commercial cell lines), even though in vivo tumor cells are surrounded by matrix cells and grow as tissues. Accordingly, scientists developed co-culture systems to rebuild cell-to-ECM (extra-cellular matrix) communication environment. However, the stromal cells (e.g., fibroblasts and endothelial cells) used in such systems are either from immortalized cell lines^4–6^ or exogenous hosts^7^. Thus, there is considerable uncertainty as to whether tumor cells in these artificial microenvironments function as they do in their native states.

Today, the most advanced in vitro cancer model is the tumor organoid^8^. Because it is derived from cancer stem cells that retain the original tumor’s heterogeneity^9^, tumor organoids have been used to study the occurrence, development and treatment of tumors^10–12^. However, the organoid model also has some limitations. The major challenge is the lack of a native microenvironment (e.g., ECM composition and growth factor gradients). Furthermore, the development of organoids relies on the addition of exogenous growth factors and cell-signaling pathway regulators, and these may change the physiological conditions significantly from the tumor’s original microenvironment. When grown in these artificial cultures, tumor organoids are prone to cell division errors—resulting in much slower growth rates than the parental tumors in vivo^2,13,14^.

An ideal in vitro model for cancer study would closely replicate a tumor-specific physiological or pathophysiological microenvironment, thereby maintaining native cell-to-cell and cell-to-ECM interactions and signal transduction paths that rely on specific tissue structures^1^. As a consequence, current pre-clinical drug-sensitivity/resistance assays are not reliable, so they are not widely used in oncology practice.

To achieve a more individualized in vitro tumor model, we used a patient’s own serum or body fluid to build the 3D scaffold and serve as the culture medium. We named this technique “3D-ACM” (autologous culture method). Traditional 3D-FBS (fetal bovine serum) cultures were used for comparisons. 3D-ACM was significantly better in preserving the parental tumor’s histopathology, immune phenotype expressions and cytokine productions. In addition, tumors in 3D-ACM were much less sensitive to chemo drugs than in 3D-FBS.

## Results

A total of forty-five clinical cancer samples (Table 1) were cultured with the 3D-ACM technique; all samples survived in autologous cultures and formed tissue-like structures therein. The culture duration for solid tumors averaged 15 days (range of 7–24 days), depending on the volume of serum donated by the patient (we usually obtained 10–20 ml whole blood). The rate of solid tumor growth was estimated by the change in tumor size under microscope, as shown in Supplementary Fig. S1a. Liquid sample cultures had longer durations—weeks or even months (over 3–4 passages), because of the large volume of body fluid that was available (we typically obtained 500–800 ml). 3D-FBS cultures were also performed for these samples, side-by-side. Compared to 3D-ACM cultures, all tumors grew more slowly in 3D-FBS (Supplementary Fig. S1b,c), and no liquid samples in FBS survived the passage process. Tumor samples from both solid tissues and body fluids contained multiple types of cells; in addition to cancer cells, there were stromal cells and infiltrated lymphocytes in solid tissues and mesothelial and various blood cells in body fluid samples (Supplementary Fig. S1d).

**Table 1:** Clinical samples.

Tumor types	Cases	F/M	Age range
**Solid tumors**^**a**^			
Lung adenocarcinoma (LC)	11 (two biopsies)	6/5	54–75
Gastric adenocarcinoma (GC)	8	3/5	40–80
Breast cancer (BC)	8 (two biopsies)	8/0	37–57
Lymph node metastatic cancer^c^	1	0/1	57
Total	28	17/11	37–80
**Body fluids**^**b**^			
Ascites			
Gastric cancer	2	0/2	46, 60
Ovary cancer	2	2/0	25, 70
Pancreatic cancer	2	0/2	39, 77
Lung cancer	1	0/1	51
Endometrial cancer	1	1/0	60
Total	8	3/5	25–77
Pleural effusion			
Lung cancer	6	4/2	36–86
Breast cancer	1	1/0	63
Gastric cancer	1	0/1	76
Malignant mesothelioma	1	0/1	58
Total	9	5/4	36–86

^a^All solid tumors were from surgical operation except four from biopsies (as labelled).

^b^Body fluids were freshly collected from the chest or abdomen cavity of patients with malignant cancer. These samples were called “liquid samples” in this article.

^c^Lymph node metastatic cancer: sample from metastatic lymph node of a gastric cancer patient.

F/M: female vs. male.

### Tissue-like structures only developed in 3D-ACM cultures

**Figure 1:**
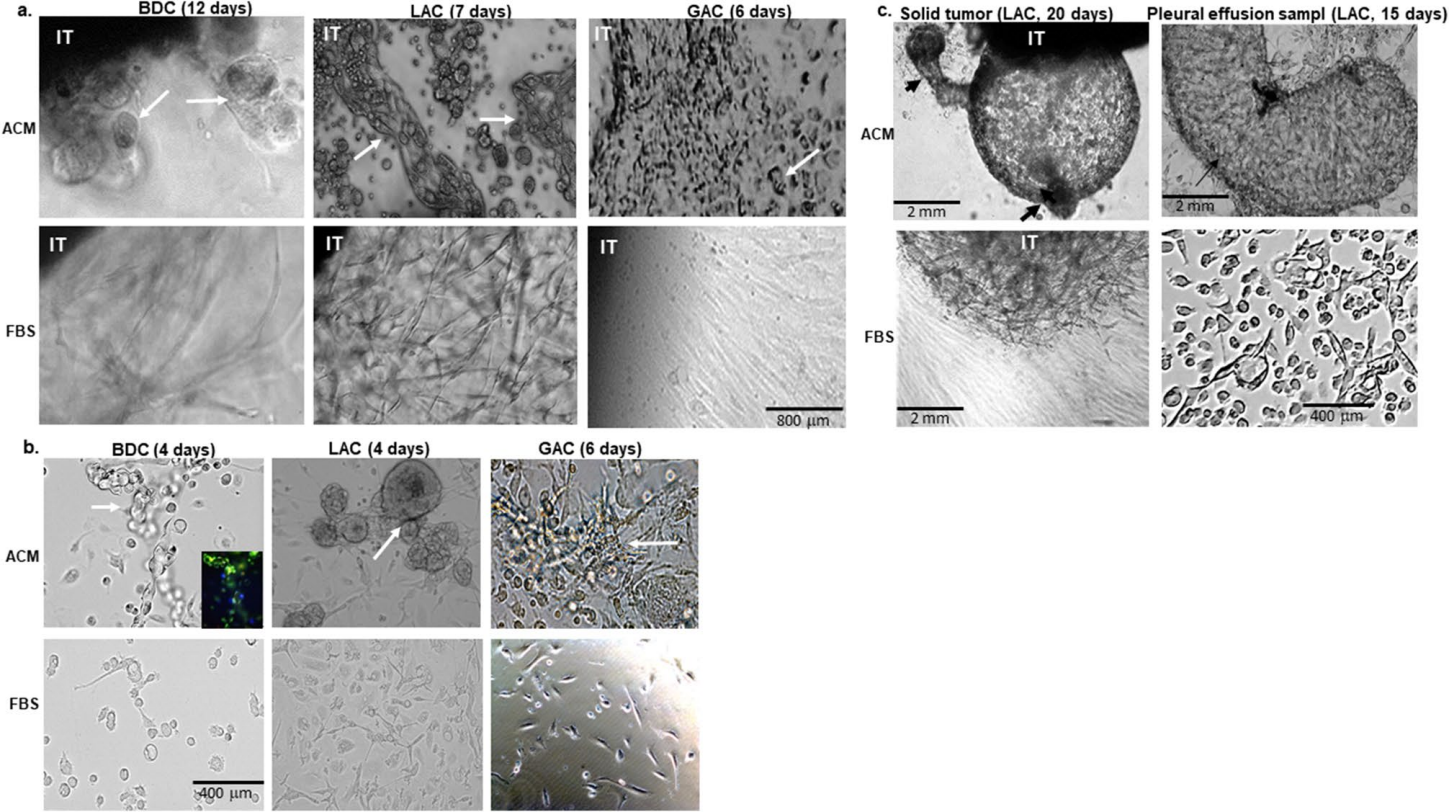
Differences between ACM and FBS in culture images. Breast ductal carcinoma (BDC), lung adenocarcinomas (LAC), and gastric adenocarcinoma (GAC) from (**a**) surgically removed solid tumors or (**b**) isolated from ascites/pleural effusion, were cultured with ACM (upper rows) and FBS (lower rows) side-by-side**.** Tissue-like structures appeared in all ACM cultures (white arrows), but not in FBS. (**c**) Masses in ACM cultures (top row) of solid LAC sample (left column) and pleural effusion LAC sample (right column) after longer culture duration (≥ 15 days); no masses in FBS. The newly formed LAC globular tumor had several new branches that budded out and formed new tumors (black arrow heads; top row on the left). IT: implanted tissue. Scale bar as indicated.

The two culture conditions, 3D-ACM and 3D-FBS (hereafter "ACM" and "FBS"), produced different growth patterns in all samples tested. Representative examples are provided in Fig. 1a,b. For solid tumors, paired images of breast ductal carcinoma (BDC), lung adenocarcinoma (LAC) and gastric adenocarcinoma (GAC) are shown in Fig. 1a. In ACM cultures, cells migrated from implanted tumors (IT) and formed globular (BDC), glandular (LAC), or trabecula with small glandular (GAC) structures (see arrows) as early as six days after implantation (upper row). However, no such structures formed when the same tissues were implanted in FBS cultures; instead, fibroblast-like cells dominated in those culture wells (lower row). Similar results were observed in liquid sample cultures (Fig. 1b), wherein mixed cell suspensions isolated from cancerous body fluids were seeded onto autologous 3D scaffolds. Self-organized, tissue-like structures—tubulars in BDC, globules in LAC and trabecula forms in GAC—appeared in ACM wells as early as four days in culture (upper row), but not in any FBS wells where fibroblast-like cells again dominated (lower row). Live/dead cells viability stain showed that the tissue-like structure that formed in the BDC sample was not simply cellular aggregation, but had a tubular architecture (see insert image of BDC in Fig. 1b). Video images showing the process of cells self-organizing in ACM are provided in Supplementary Videos 1 and 2. Video 1 shows a gastric adenocarcinoma growing in ACM: when large, round cells (possibly epithelial cells) migrated out from the implanted tissue, a connective tissue-like structure (composed of crossed spindle cells) followed behind. Video 2 shows isolated cells from a LAC in ACM culture. After self-organization, single cells in the suspension contacted each other and formed a net. To better compare the differences in growth patterns between ACM and FBS, daily images showing changes of tissue or cells in these two cultures are provided in Supplementary Fig. S2. The differences in growth patterns between ACM and FBS cultures became more obvious with longer culture durations (≥ 15 days). Cancer cells from both solid and liquid samples formed tumor masses in ACM—but not in FBS, where the domination of fibroblast-like cells continued (Fig. 1c).

**Figure 2:**
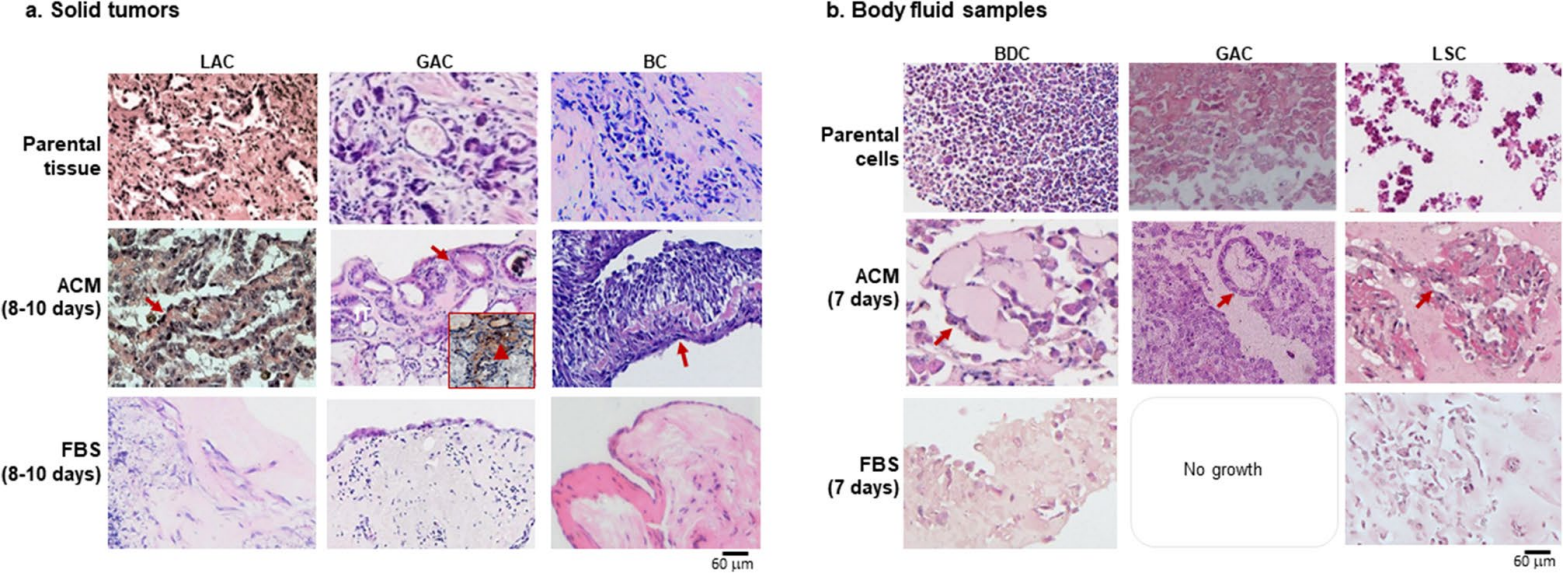
The similarity in histopathology of ACM-cultured tumors to parental cancers. Hematoxylin and eosin (H&E) stains. (**a**) Solid tumors; (**b**) Body fluid samples. Histopathology of different tumors: parental (top row), ACM cultures (middle row), and FBS cultures (bottom row) in (**a**) and (**b**) panels. Tissue-like structures formed in all ACM cultures (red arrows). The insert in ACM-cultured GAC shows that the newly formed glands were positive to PCNA, which means cells were still proliferating after 8–10 days in culture (red arrowhead). LAC: lung adenocarcinoma, GAC: gastric adenocarcinoma, BC: breast invasive ductal carcinoma, LSA: lung squamous cell carcinoma. Scale bar = 60 µm.

In hematoxylin and eosin stains, the newly formed structures in ACM resembled the histopathology of their parental cancers in both solid tumors (Fig. 2a) and body fluid samples (Fig. 2b). In the solid tumors, histopathology showed irregular glands in both LAC and GAC parental tissues. Similar glandular structures formed in ACM cultures. The BDC is a breast-invasive ductal carcinoma with no glandular or tubular structures in the original tumor by histopathology. In ACM culture, this sample grew with non-specific structures. In the body fluid samples, although these cultures were initiated with liquid cellular suspensions, tubular structures were observed in BDC, glandular structures were found in GAC, and cellular nests appeared in LSC (lung squamous carcinoma). In contrast, in FBS cultures, almost all original structures in solid tumor implants disappeared; only a few matrix cells remained in cultures of both solid and liquid samples (Fig. 2a,b).

Replacing autologous culture media with other types of human sera in our 3D cultures had a negative impact on tumor growth. The use of commercial human serum resulted in tumor death within three days (n = 3; solid tumor tissues). Using exogenous culture medium (ECM) to replace ACM produced slower tumor growth or cell death: In breast cancer cultures (solid tumor sample), two ECMs (sera from two different breast cancer patients) that were used individually caused slower growth of the implants relative to ACM (Supplementary Fig. S3a). In LAC cultures, three ECMs (pleural effusions from three different LAC patients) were used to grow a LAC tumor individually. More cell death occurred, and fewer tissue-like structures formed in the ECM cultures than in ACM (Supplementary Fig. S3b).

### ACM better-maintained immuno-phenotypes in new growths of cancer tissues and cells

**Figure 3:**
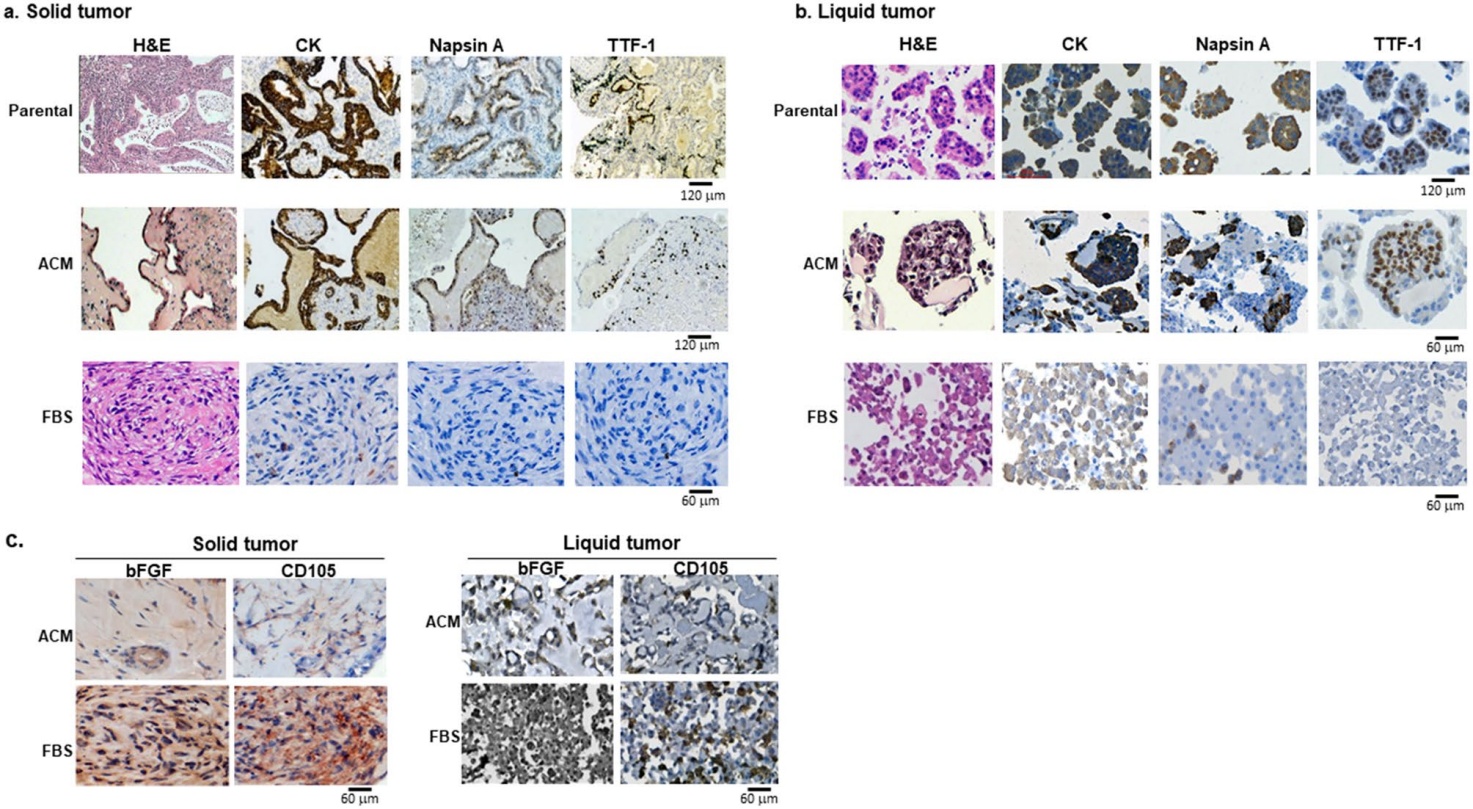
The similarity in immune phenotypes of ACM-cultured tissues to parental cancers. H&E and IHC stains of lung adenocarcinomas. (**a**) Solid tumor; (**b**) Pleural effusion sample. Parental tumors (top row; scale bar = 120 µm), ACM-cultured (middle row; scale bar for solid tumors is 120 µm, and for body fluid samples is 60 µm) and FBS-cultured (bottom row; scale bar = 60 µm). Antibodies: CK (cytokeratin), Napsin A and TTF-1. (**c**) IHC for FGF and CD105 expressions in above cultures (scale bar = 60 µm).

Immunohistochemistry (IHC) was performed for lung cancers (n = 13), breast cancers (n = 4) and gastric cancers (n = 2). Parental immune phenotypes were retained much better in ACM cultures than in FBS. Representative results for LAC are shown in Fig. 3. The markers that are routinely used by pathologists for LAC diagnoses—CK, Napsin-A and TTF-1—were well-expressed in new growths of LAC in ACM, for both solid (Fig. 3a) and liquid samples (Fig. 3b). However, in FBS cultures these markers were either negative (in solid tumors) or poorly expressed (in liquid samples). The similarity of a new growth to its parental gastric adenocarcinoma (ascites sample) is shown in Supplementary Fig. S4. IHC studies, using Abs against bFGF and CD105, indicated that the dominant cells in FBS cultures were mainly mesenchymal. Almost all cells in the FBS cultures were strongly positive for these two markers. However, in ACM-cultured tissue, they were only detected in the matrix areas between tumor nests (Fig. 3a,b).

### Growth factors in culture media were different between ACM and FBS

**Figure 4:**
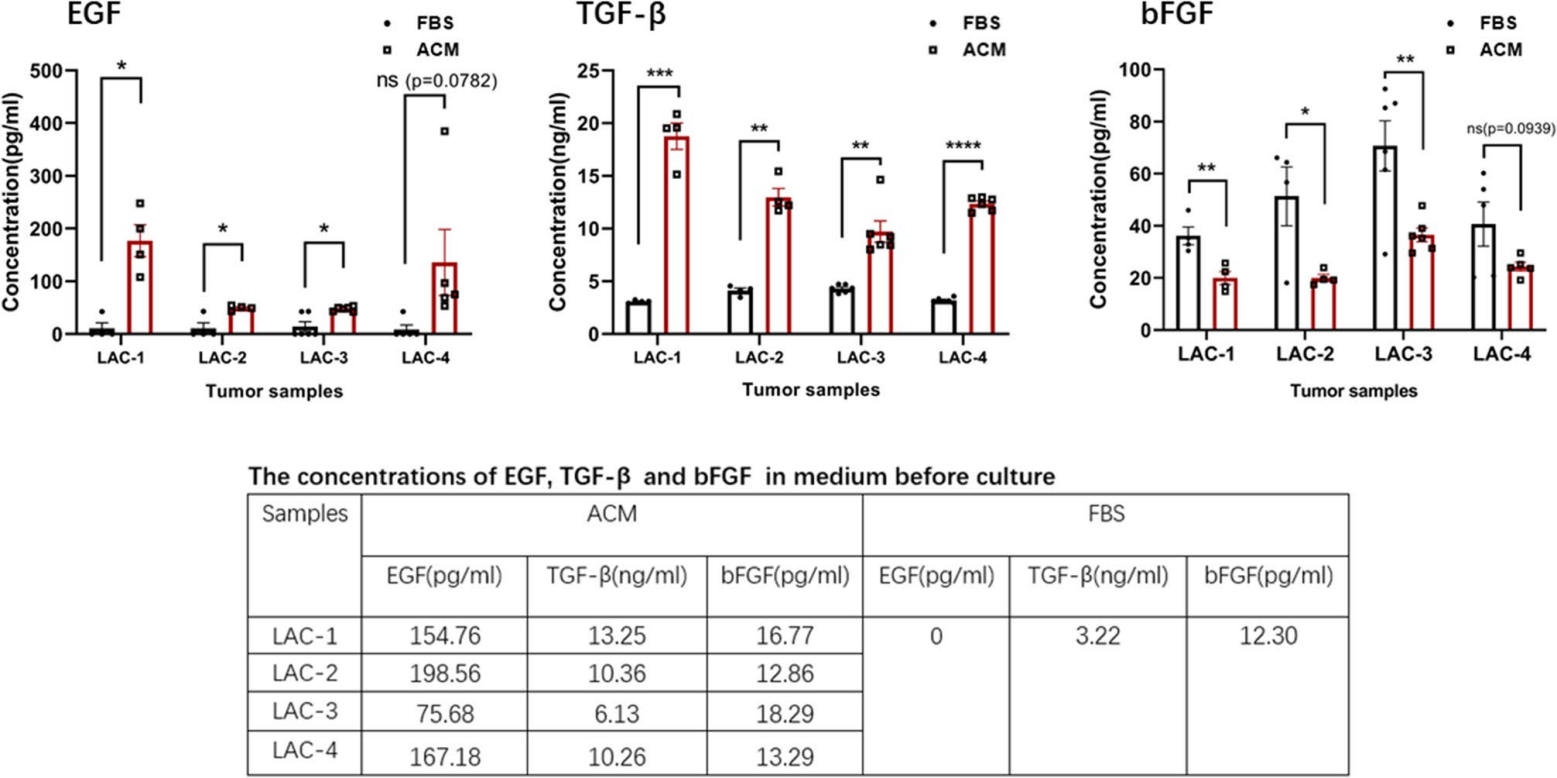
Differences in growth factors between ACM and FBS cultures. ELISA for EGF, TGF-b and bFGF concentrations in media from four LAC (pleural effusions) cultures. Media were collected after 10–15 days in culture. Concentrations are means of 4–6 wells/sample. The concentrations are shown in the columns: red for ACM and black for FBS. The numbers of culture wells/sample are indicated by square dots for ACM and by triangles for FBS. Inserted table shows the concentrations of these growth factors in intact ACM and FBS media (the FBS medium was the same for all culture wells). The difference between ACM and FBS for an individual sample was analyzed with student *t*-test: * < 0.05 , **p < 0.01 , ***p < 0.001, and ****p < 0.0001. The differences between ACM and FBS as a group for each growth factor were also analyzed with one way ANOVA, *p* ≤ 0.001 for all three groups. Tests were repeated 2 or 3 times, with similar results.

To understand the mechanism for the different morphologies described above, using Enzyme-Linked Immunosorbent Assays (ELISA), we compared the concentrations of EGF (epidermal growth factor), TGF-β (transforming growth factor beta), and bFGF (basic fibroblast growth factor) between the ACM and FBS media. Four pleural effusion samples of LAC were analyzed; the results are shown in Fig. 4. After 10–15 days in culture, the EGF and TGF-β in ACM-culture media were both significantly higher than those in FBS. The average concentration of EGF in ACM was 82.67 ± 13.96 pg/ml, while in FBS it was 11.29 ± 4.45 pg/ml. For TGF-β the average level in ACM was 12.96 ± 0.83 ng/ml, but in FBS it was 3.69 ± 0.14 ng/ml. In contrast, higher bFGF concentrations were detected in FBS (51.26 ± 5.32 pg/ml) than in ACM (26.33 ± 1.97 pg/ml). Using one way ANOVA analysis, significant differences between ACM and FBS were found (*p* ≤ 0.001) in most samples for the three growth factors. The insert table in Fig. 4 provides the concentrations of these cytokines in the intact media (i.e., before cultures). There was no EGF and significantly lower TGF-β (3.22 ng/ml) in the original FBS, but these two cytokines were detected in all four fresh ACM media, 149.04 ± 26.14 pg/ml for EGF, and 10.00 ± 1.46 ng/ml for TGF-β. The bFGF was nearly the same in the original ACM and FBS media (15.30 ± 1.33 vs. 12.3 pg/ml). We also compared the concentrations of EGF, TGF-β, and bFGF between the ACM and FBS media from solid tumor cultures of breast cancers (n = 6). Similar to the results for pleural effusion of LAC, significantly higher levels of EGF and TGF-β were detected in ACM before and after cultures. But in FBS, the EGF was absent and the level of TGF-β was much lower before and after cultures (Supplementary Fig. S5a,b). The bFGF in ACM and FBS were similar in the intact media; for these breast cancer samples, an elevated concentration was only detected in FBS (Supplementary Fig. S5c).

### Tumors in ACM were more resistant to chemotherapy drugs

**Figure 5:**
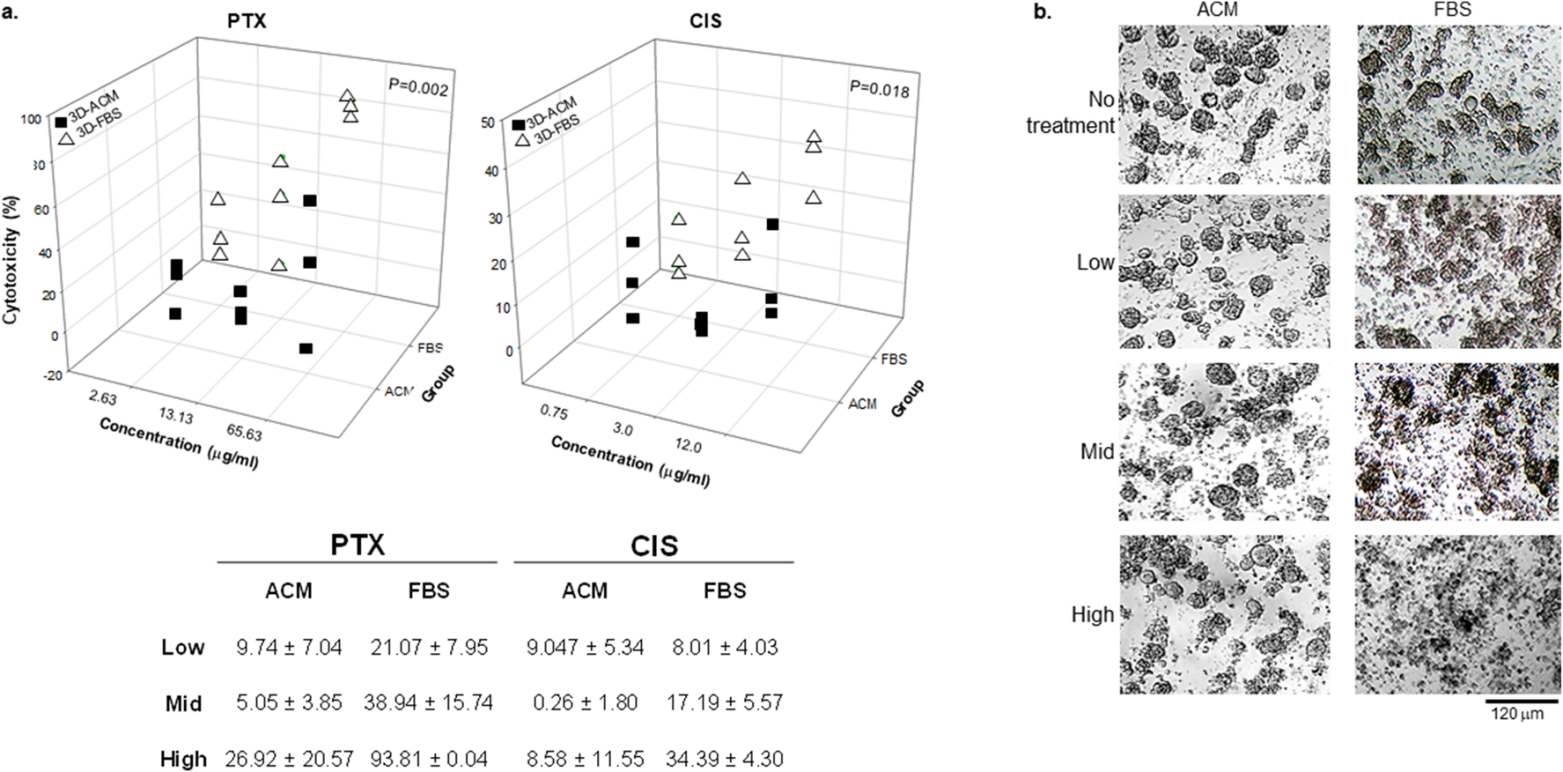
Differences in drug-sensitivity between ACM- and FBS-cultured cells. (**a**) Cytotoxicity to Paclitaxel (PTX) and Cisplatin (CIS) measured with CCK-8 kit for three lung cancers (pleural effusions). Each drug was applied in low, medium, and high concentrations. The cytotoxicity (%) was calculated using the formula provided by the manufacture. ∆ represents FBS cultured cells, ■ represents ACM. Inserted table shows the mean cytotoxicity for a concentration; p-values are from two-way ANOVA test. (**b**) Cell morphologies under ACM and FBS cultures, with or without PTX treatment, at 24-h time-point (Scale bar = 120 µm). This assay was repeated 3 or 4 times with similar results.

To investigate whether the morphological differences in ACM and FBS cultures had any impact on cellular behaviors, drug-sensitivity assays were performed on three pleural effusion samples of LAC. Freshly isolated cell suspensions were stabilized in ACM and FBS culture wells for 24 h before drugs were applied. The chemo drugs Paclitaxel (PTX) and Cisplatin (CIS) were used in these assays; both are recommended by the NCI (National Cancer Institute) for non-small cell lung cancer. Two-way ANOVA analyses indicated that, for all three samples, cells under the FBS condition were much more sensitive to both PTX (*p* = 0.002) and CIS (*p* = 0.018) than in ACM cultures. No obvious differences in results were found between samples incubated for 24- or 48-h with these drugs. The cell toxicities at the 24 h time-point are shown in Fig. 5a. Morphologically, cell spheres can be seen in ACM cultures for all three concentrations of PTX, but they became fragmented or resembled debris in FBS, even at the lowest concentration (Fig. 5b).

## Discussion

This study demonstrated the effects of using ACM for primary tumor cultures, as compared to the traditional FBS technique. ACM provides an ecosystem for tumor cells that is very similar to their native condition, including the 3D environment and autologous culture medium (≥ 50% for autologous serum and 100% for autologous body fluids). No commercial or exogenous bio-reagents are used in the ACM cultures because the culture environment, prepared with autologous serum or body fluids, contains all the nutrients, hormones, cytokines/chemokines and growth factors that an individual tumor needs—and at their in vivo physiological concentrations. With commercial products, it would be nearly impossible to provide such an individualized ecosystem for a patient’s tumor. ACM maximally preserved the natural heterogeneity of a tumor and its surrounding tissues, cells and matrix—including some infiltrated lymphocytes (Supplementary Fig. S1d). No enzyme digestion was employed for either solid or liquid samples, nor were particular cell populations selected before cultures. A solid tumor was implanted in culture as tissue pieces, such that the original microenvironment of the tumor was well-preserved in vitro. For body fluids, all cell-types (except red blood cells) in the original liquid were seeded onto the autologous 3D scaffold, which enabled all of them to participate in the reconstruction of tissue-like structures. The results of our experiments were that tumor cells quickly adapted to the ACM environment, rebuilt the histological structures (Figs. 1and 2), retained the immune phenotypes (Fig. 3) and cytokine productions (Fig. 4), and possibly preserved the drug sensitivities of the original tumors (Fig. 5).

Our study also revealed that the FBS-complemented 3D technique for primary cultures mainly encouraged mesenchymal cell proliferation. In all FBS cultures, fibroblast-like cells were dominant and strongly positive to bFGF and CD105 (Fig. 3C). In addition, no tissue-like structures were observed in any FBS-cultured solid or liquid samples.

We also varied the type and concentration of serum used in some of our cultures. Commercial human serum was employed in two 3D cultures of solid tumors; the tumors in these cultures died within three days. Different concentrations of autologous serum—10%, 25%, 50%, and 100%—were tested in three breast cancer samples for their effects on tumor growth. The results were that a higher concentration of autologous serum produced faster tumor growth combined with better-formed tissue-like structures. Varying the concentration of FBS-complemented culture medium—100%, 50% and 10%—did not show morphological differences in the cultured cells. Due to the limited number of samples, the above data are not presented in this report. The drawbacks we observed when using exogenous sera or body fluids in the cultures (Supplementary Fig. S3a,b) further confirmed the advantage of the ACM technique for maintaining a tumor’s biological characteristics.

The differences in growth factor productions between ACM and FBS media may be at least partially responsible for the differences in growth patterns and morphologies between these two cultures. The EGF and TGF-β levels were significantly higher in all ACM media before and after cultures, relative to in FBS, which had no EGF and very low TGF-β. EGF is a ligand for the EGF receptor (EGFR). EGF and EGFR control important processes in carcinogenesis, and this signaling pathway is very common in certain types of cancers^15,16^. The TGF-β family proteins possess a relatively complex nature. TGF-β plays a predominant role in epithelial-mesenchymal transition (EMT)^17^. Previous reports also indicate that TGF-β is a bifunctional regulator that can either promote or inhibit growth of the same cell type, depending on the experimental conditions^18^. Some evidence supports the view that increased TGF-β expression in cancer promotes tumor progression by enhancing migration, invasion and the survival of tumor cells during tumorigenesis^19^. A later study by Hao et al. found that TGF-β acted as a tumor suppressor during the early stages of tumorigenesis, although at later stages it functioned as a tumor promoter by stimulating cancer cells to undergo EMT, and by activating tumor angiogenesis and cancer-associated fibroblasts^20^. We were unable to determine the functions of these cytokines in our ACM cultures, so that we refrain from drawing any conclusions as to their effects on our results. Further investigation is needed to fully understand the roles of EGF and TGF-β in primary cultures of cancer tissue.

Among the three growth factors tested, only bFGF had a higher level in FBS after cultures. FGF was originally identified as a protein capable of promoting fibroblast proliferation, and is a growth factor having potential effects on wound repair and tissue regeneration^21^. Accordingly, FGFs are utilized for the regeneration of damaged tissues, including skin, blood vessel, muscle, adipose, tendon/ligament, cartilage, bone, tooth, and nerve^22–25^. In all our FBS cultures, uniform fibroblast-like cells dominated and, unlike the results with ACM, there were no tissue-like structures. Based on our culture images, histopathology and IHC results, we surmise that primary cultures using FBS medium promote the growth of mesenchymal cells, rather than epithelial cancer cells.

The differences in morphology and biochemistry between ACM and FBS cultures correlated with differences in the biological behaviors of the tumor cells. In our preliminary drug sensitivity assays, a much higher toxicity was associated with FBS-cultured cells at the tested concentrations of the two drugs employed. This result might be due to the failure of FBS to fully satisfy nutritional and other physiological needs, such that tumor cells in FBS were not as healthy as those in ACM when the drugs were applied. In addition, cells in FBS lost the ability to form tissue-like structures, so they were more directly exposed to the chemo drugs. In contrast, ACM cultures provided the tumor cells with an environment that was much closer to their native condition, which apparently made them more robust and enabled them to grow together into their original form. Accordingly, with ACM culture, we expect drug sensitivity results to more closely represent clinical outcomes. Using autologous serum (20%) to grow primary tumors for drug sensitivity/resistant assays was first reported in 1961 under a 2D culture condition^26^. In that study, Dr. Cobb et al. found that tumors survived much better in the autologous serum-complemented medium than in heterologous (horse) or homologous (pooled normal human) serum-complemented media. Consistent with our results, they also observed reduced sensitivity to chemo drugs with the autologous serum, as compared to the other sera. However, under their 2D conditions, no tissue-like structures formed in their autologous cultures.

Interestingly, unlike the assays in our FBS cultures, the cellular toxicities in ACM were not proportional to the drug concentrations (Fig. 5 insert table). The drug-sensitivity assays were repeated 3–4 times and, in all ACM cultures, the low doses induced higher cytotoxicity than the medium doses. We speculate that this might be due to the greater variety of cell-types that survived in ACM cultures, but a definitive explanation must await further studies.

The rearrangement and self-organization of cells into tissue-like structures were observed in all ACM cultures. We noted that some of these structures resembled “organoids” as described in some previous studies^27–30^. However, the organoid-like structures in our ACM cultures did not rely on any additional growth factors or gene inducers. Instead, the autologous serum or body fluid enabled spontaneous self-organization of the tumor cells and their own surrounding matrix cells. In our experiments, ACM-cultured cells generally formed structures within 3–5 days (liquid samples) and grew into tissue-like structures or tumor masses after about 7–10 days (solid tumor tissues). This is much faster than current organoid cultures from stem cells, which typically take weeks, or even months, to grow such structures^31^. In general, clinical drug-sensitivity assays need to be timely, as well as inexpensive and reliable. The ACM appears to have the potential to meet these requirements.

To summarize, we developed a novel culture technique that is based on the use of autologous serum or body fluids in a 3D condition. This method provided an autologous ecosystem for primary cancer cultures and resulted in the reliable and rapid growth of a variety of individual cancers in vitro. Biological characteristics of the parental cancers were much better-retained in ACM, as compared to traditional FBS cultures or other non-autologous media. Preliminary drug tests indicated that cancer cells were much more vulnerable to chemo drugs in FBS cultures than in ACM. Accordingly, the 3D-ACM technique may have potential for improving the accuracy of clinical drug-sensitivity assays.

## Methods

### Collection of clinical samples

Patients' samples were provided by three hospitals during 2015–2018: Dalian Municipal Central Hospital, Dalian, China; Cancer Hospital of Shantou University Medical College, Shantou, China, and Shantou Central Hospital, Shantou, China. The study was approved and monitored by the Research Ethics Committees of Dalian Municipal Central Hospital (YN2014-023–01), the Research Ethics Committees of Cancer Hospital of Shantou University Medical College (YN201728), and the Research Ethics committees of Shantou Central Hospital (KY2018010) respectively. All procedures were carried out in accordance with relevant guidelines and regulations, and informed consent was obtained from all participants. The clinical materials included 28 solid tumors (24 surgically removed cancers and 4 biopsies) and 17 cancer-induced body fluids: ascites (n = 8) and pleural effusion (n = 9), with no limitations on sex or age (Table 1). Tumors were from eight different organs or tissues: lung (n = 18), stomach (n = 11), breast (n = 9), pancreas (n = 2), ovary (n = 2), lymph node (metastatic gastric adenocarcinoma; n = 1), endometrial membrane (n = 1) and pleural mesothelium (n = 1) (Table 1). Tumor diagnoses were confirmed by pathologists from participating hospitals. Patients who had undergone chemotherapy within three months prior to sample collection were excluded.

### Autologous cultures

The detailed procedure for autologous cultures is illustrated in Supplementary Fig. S6. For solid tumor cultures, the autologous medium (AM) was the patient’s serum. Generally, 10–20 ml of fresh blood was collected from each patient. This yielded 5–10 ml of serum, which was then 1:1 diluted in RPMI-1640 (50%). The AM for body fluid samples was pure ascites/pleural effusion (100%), which was first centrifuged at 2000 rpm for 10 min to precipitate cells, then filtered through a 0.45µ filter-unit (Fisher Scientific, Cat# 09-740-24B, USA). To prepare the autologous 3D scaffold, Matrigel (BD Biosciences, Cat# 356243, USA) was well-mixed with 100% autologous serum (AS) or body fluid at a 1:1 ratio; we named this "AM-matrigel".

Solid tumor tissues were cut into pieces (≤ 0.5 mm in diameter), then placed onto AM-matrigel pre-coated wells at 3–4 pieces/well in a 12- or 24-well plate; 4–6 wells were used per tumor sample (Supplementary Fig. S6a). After incubation at 37 °C for 30 min, these tissues were covered with AM-matrigel to complete the 3D environment. AM was then added into the well after the AM-matrigel polymerized. For liquid sample cultures, the cell-pellet from the original spin (see above) was loaded onto Percoll gradient centrifugation solution (TBD, China, Cat# LTS0770125) to remove red blood cells. Cells from the enriched layer were collected and washed in PBS twice, then re-suspended in autologous body fluid. They were then counted and seeded in the AM-matrigel pre-coated wells at 1 × 10^6^ in 10 ml per 100-mm dish, and 5 × 10^5^ in five ml per 60-mm dish (Supplementary Fig. S6b). All AM and AM-matrigel contained Cefoperazone (Pfizer Dalian Pharmaceutical Plant, China) at a final concentration of 20 µg/ml. To maintain a multicellular condition, only half of the AM was refreshed 2–3 days following the start of the culture. After all cells either adhered or self-organized in the culture well, the entire volume of AM was refreshed every 3–4 days.

### Cultures used for controls

For comparison purposes, FBS (fetal bovine serum)-complemented 3D cultures were performed side-by-side with the autologous cultures. For the FBS cultures, the “AM-matrigel” was replaced with FBS-matrigel (matrigel 1:1 diluted with 20% FBS medium) and autologous culture medium was replaced with 10% FBS-complemented RPMI-1640 medium. Both the FBS-matrigel and -medium contained Cefoperazone at the same concentration as in the 3D-ACM cultures. This procedure was named “3D-FBS” culture. In addition, normal human serum (Sigma-Aldrich, USA) and exogenous serum or body fluids—collected from different patients having the same type of cancer—were also used in some tumor cultures for comparison to ACM. We called this ‘exogenous culture medium’ (ECM). A procedure similar to that using FBS was followed in preparing these cultures.

### Tissue culture harvest

Cells and tissues were harvested when a well or dish was more than 80% confluent with new growths, or when no more AM was available. Tissues/cells were then scraped from culture wells and were either frozen at – 80 °C or fixed with 4% paraformaldehyde for future use.

### Histopathology and immunohistochemistry (IHC)

Partial parental solid tumor tissue and a small amount cells freshly isolated from body fluid (see Autologous culture section) were directly fixed in 4% formalin. Tissues/cells from culture wells were washed with PBS solution and spun at 1000 rpm for 10 min before fixation. Routine paraffin embed, slide section and hematoxylins/eosin (H&E) stain were performed for all samples tested. DAKO Autostainer Link48 was utilized for IHC stains following the company’s instructions. The antibodies employed included: CK (AE1/AE3) and TTF-1 (8G7G3/1) from DAKO Agilent Pathology Solutions (Santa Clara, CA US); Napsin-A (ZM11) and CEA (12/140/10) from ZETA Corporation (CA, USA); CD105 and Calretinin (CR) from Abcam (Cambridge, MA USA); bFGF from Abgent (San Diego, CA USA) and PCNA (Proliferating Cell Nuclear Antigen) from ZSGB-Bio (ZM-0213, Beijing, China). The second antibody used was a poly-horseradish peroxidase anti-mouse/rabbit IgG detection system (PV-9000, ZSGB-Bio, Beijing, China).

### ELISA

ACM and FBS media were collected individually from 4–6 wells of each tumor sample on the same day after culture. Corresponding intact media (before culture) were first stored at – 80 °C then tested simultaneously with media post culture. ELISA kits for human TGF-β1, EGF and bFGF (Biotech Co. Ltd, Beijing, China) were used following the manufacturer’s instructions.

### Drug-sensitivity assay

Two chemo drugs, Paclitaxel (PTX) and Cisplatin (CIS), were purchased from Hospira Australia (VIC3170; Australia) and Haosen Pharmaceutical (Lianyungang, China) respectively. Each drug’s toxicity was pre-tested in two cancer cell lines (A549 and MCF7) using serial dilution, starting with a dose close to clinical intravenous use. A concentration close to the IC_50_ results from cell line tests was used as the medium dose, then the high and low doses were calculated by multiplying or dividing the medium concentration by a factor of 4–5. Fresh pleural effusion was obtained from a patient with lung adenocarcinoma (LAC). Cells were then isolated from the effusion and seeded immediately into wells pre-coated with AM-matrigel or FBS-matrigel (see Autologous Cultures above) in a 96-well plate, at 1 × 10^5^/ml, 100 µl/well. After cells had stabilized in culture for 24 h, drugs of three different dosages were added into the corresponding wells in triplicate per dose, and the culture was incubated another 24 or 48 h. Cytotoxicity was measured with CCK-8 kit (Dojindo Co. LLC, Shanghai, China) and calculated with the formula provided by the manufacturer. This assay was repeated 3–4 times with similar results.

### Imaging system

Cell/tissue growth was imaged every two to three days using microscopy (AE2000, Motic, China). For some samples, motion pictures were recorded with CytoSMART™ (Lonza, USA).

### Statistical analyses

The multiple comparison procedures for drug-sensitivity assays were performed using the two-way ANOVA method, employing SigmaPlot software. The quantitative data for ELISA were analyzed with both student t-test and one way ANOVA analysis, and graphed by GraphPad Prism 8.0. Data were presented as mean ± SEM (standard error), and a value of *p* ≤ 0.05 was considered significant.

### Ethics issue

The study was approved and monitored by the ethics committees of the hospitals in China that participated. These are the Research Ethics Committee of Dalian Municipal Central Hospital (YN2014-023-01), the Research Ethics Committee of Cancer Hospital of Shantou University Medical College (YN201728), and the Research Ethics committee of Shantou Central Hospital (KY2018010).

## Supplementary information

Supplementary Figure 1.

Supplementary Figure 2.

Supplementary Figure 3.

Supplementary Figure 4.

Supplementary Figure 5.

Supplementary Figure 6.

Supplementary Legends.

Supplementary Video 1.

Supplementary Video 2.
