# Supplementary figures and images for: Autologous culture method improves retention of tumors’ native properties
Source: Sci Rep. 2020 Nov 24;10:20455. doi: 10.1038/s41598-020-77238-0 (PMC7686378; doi:10.1038/s41598-020-77238-0)

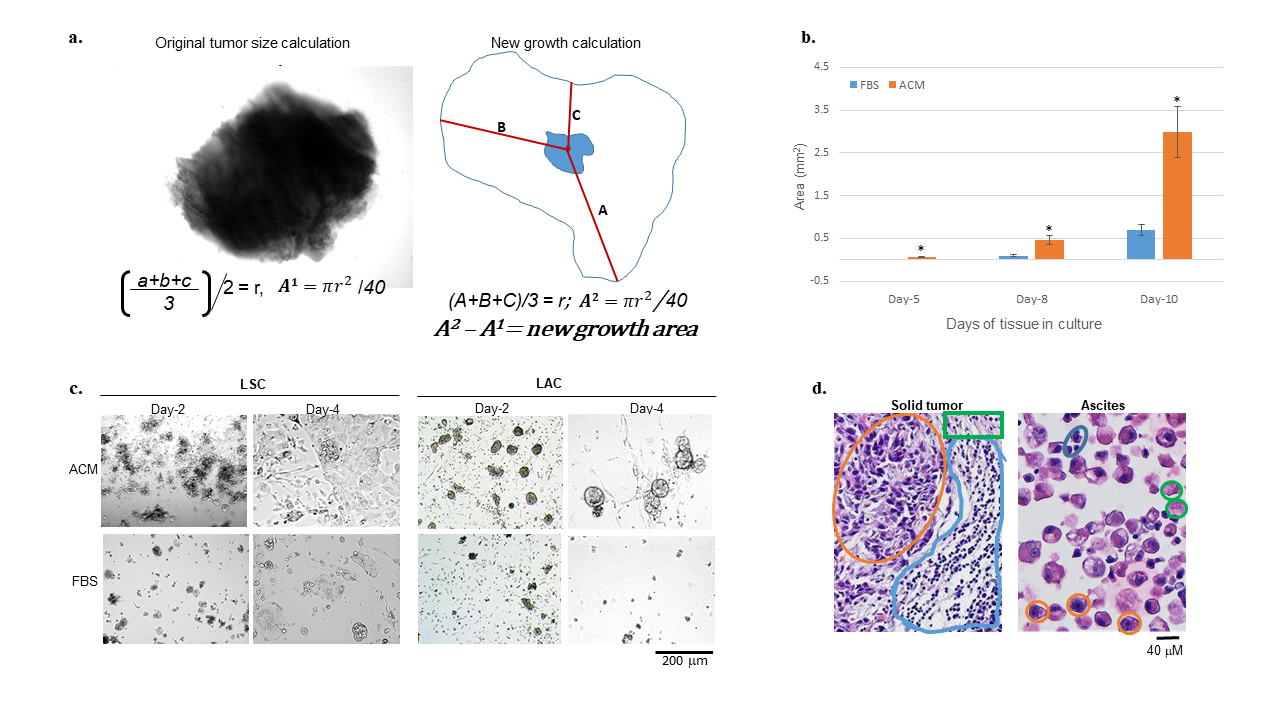

Supplement: Supplementary file 1 — Supplementary Figure 1. [file 41598_2020_77238_MOESM1_ESM.tif]

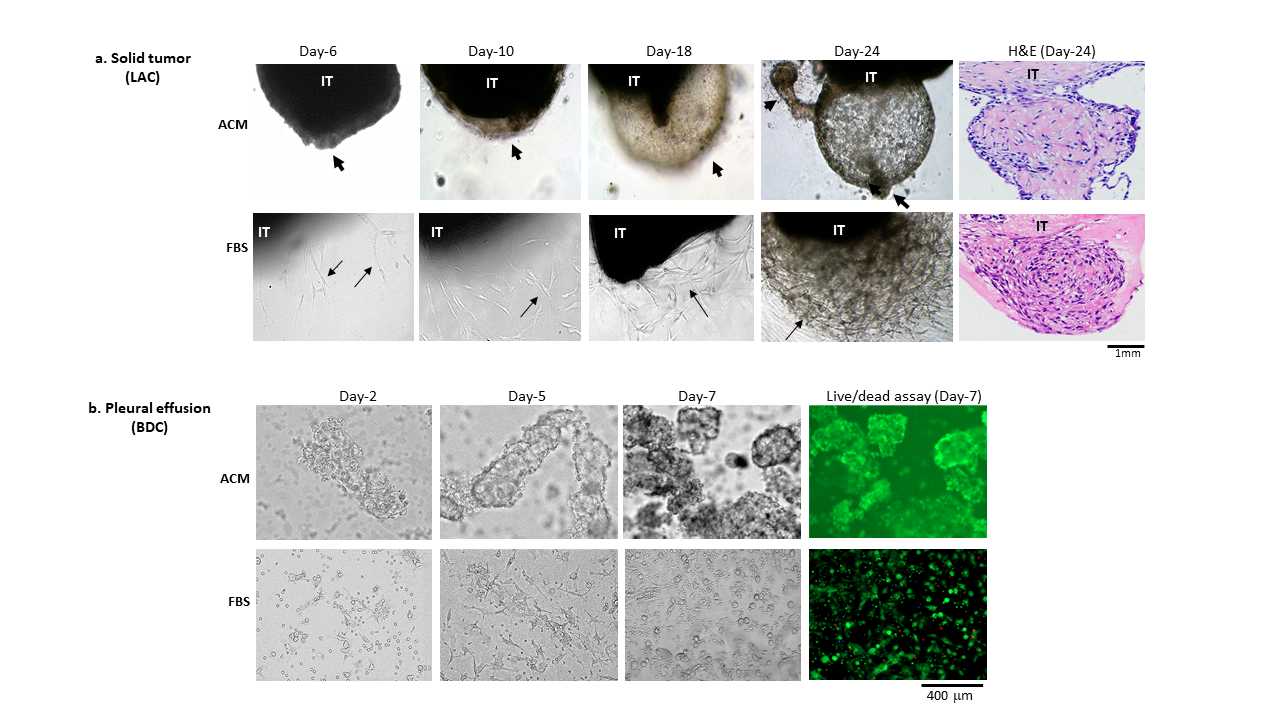

Supplement: Supplementary file 2 — Supplementary Figure 2. [file 41598_2020_77238_MOESM2_ESM.tif]

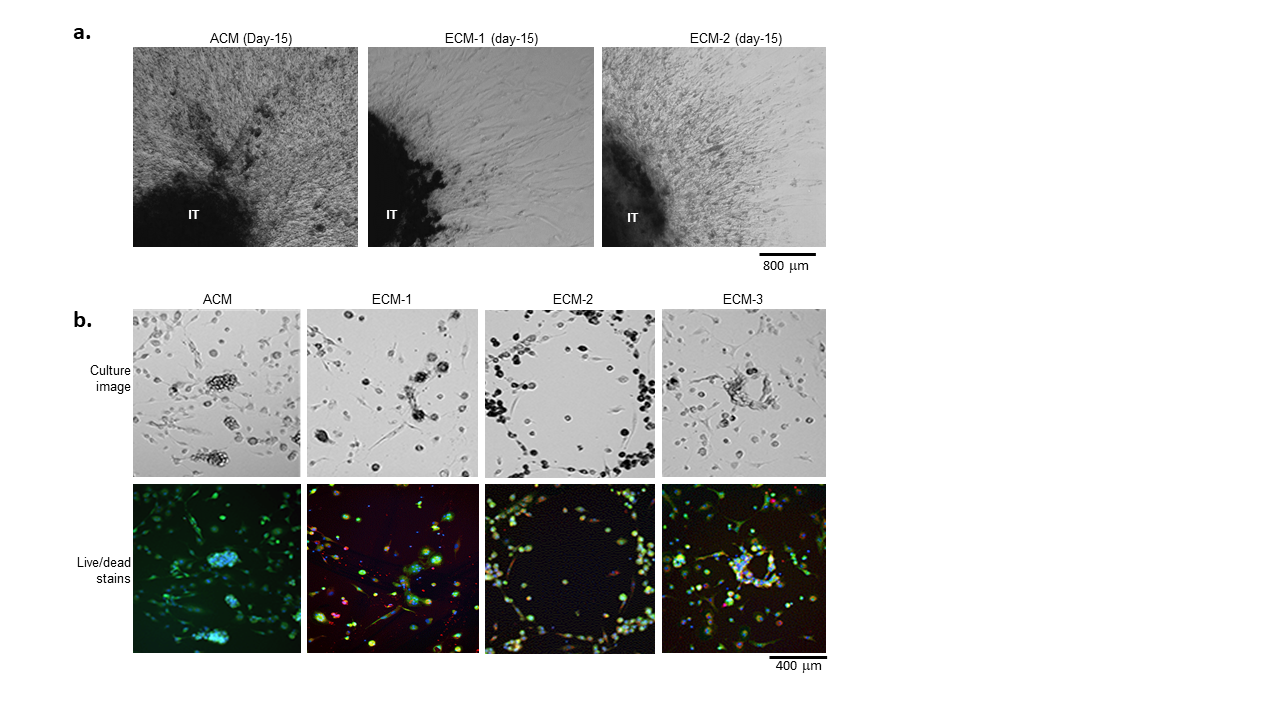

Supplement: Supplementary file 3 — Supplementary Figure 3. [file 41598_2020_77238_MOESM3_ESM.tif]

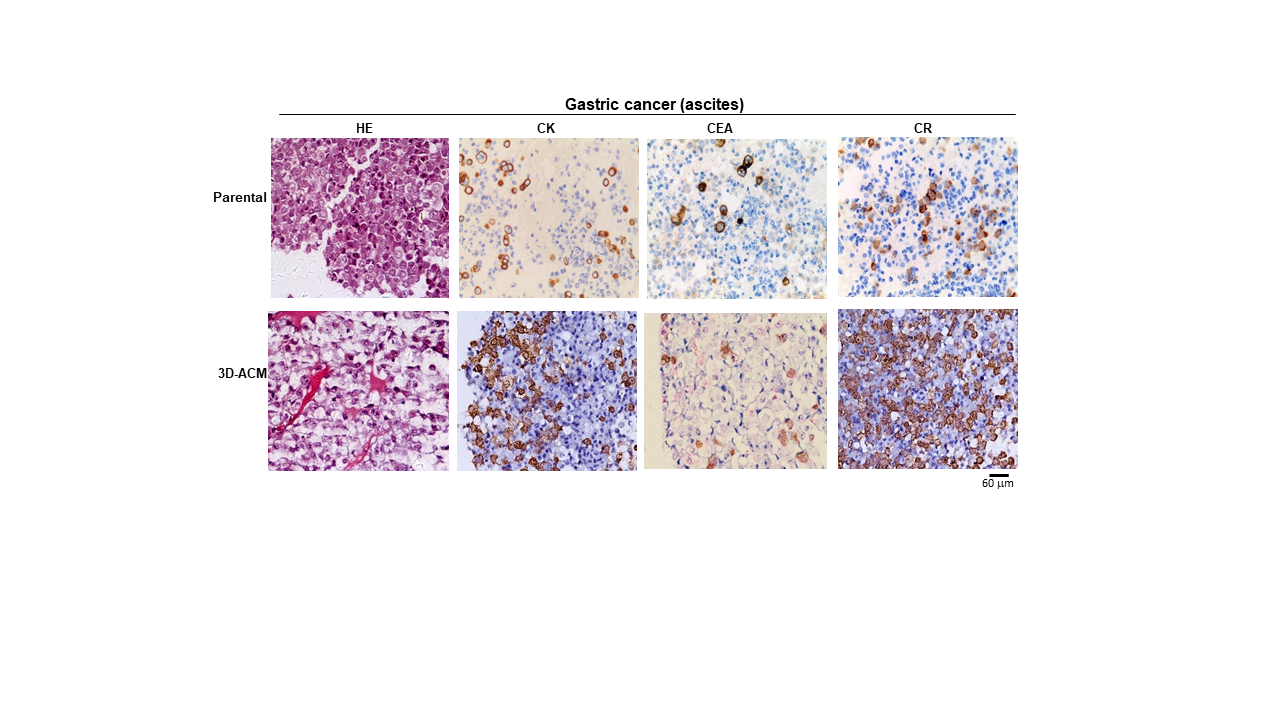

Supplement: Supplementary file 4 — Supplementary Figure 4. [file 41598_2020_77238_MOESM4_ESM.tif]

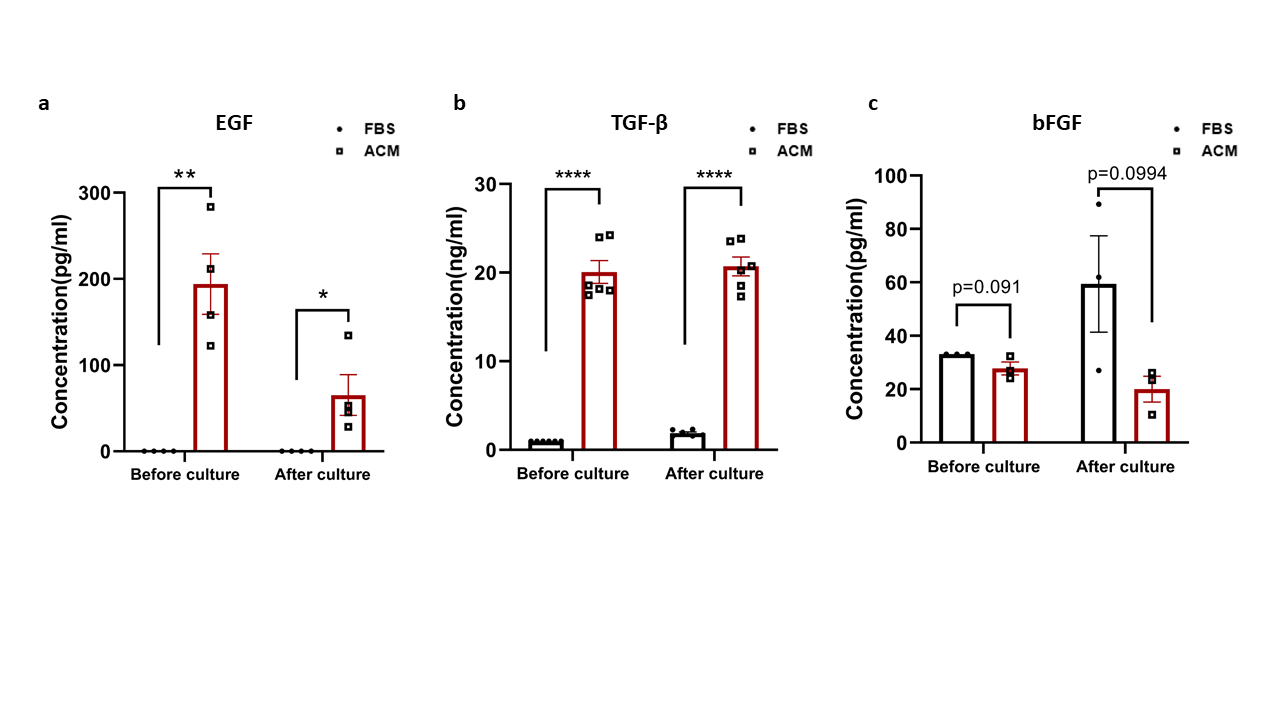

Supplement: Supplementary file 5 — Supplementary Figure 5. [file 41598_2020_77238_MOESM5_ESM.tif]

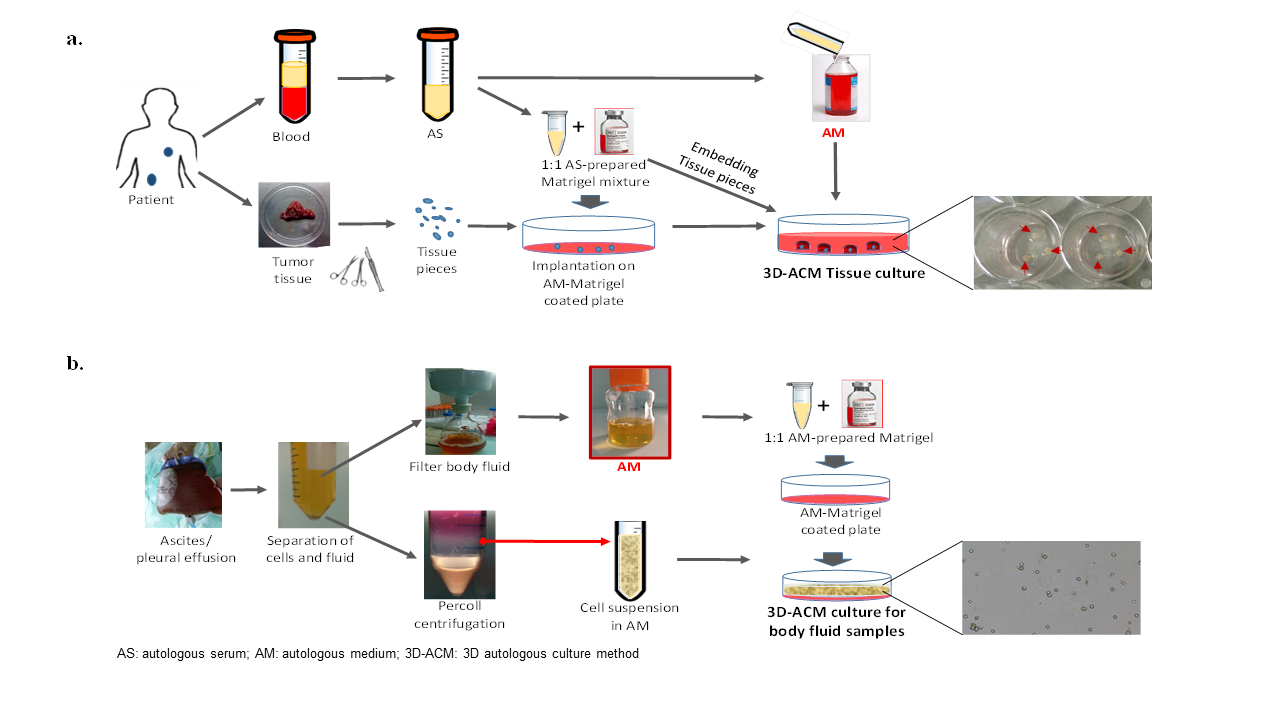

Supplement: Supplementary file 6 — Supplementary Figure 6. [file 41598_2020_77238_MOESM6_ESM.tif]
