# Supplementary material for: Autologous culture method improves retention of tumors’ native properties
Source: Sci Rep. 2020 Nov 24;10:20455. doi: 10.1038/s41598-020-77238-0 (PMC7686378; doi:10.1038/s41598-020-77238-0)
Supplement: Supplementary file 7 — Supplementary Legends. [file 41598_2020_77238_MOESM7_ESM.docx]

**Supplementary materials for manuscript titled:**

**Autologous culture method improves retention of tumors’ native properties**

*†Yao Tang^1,3^, †Qian Xu^1^, Meiling Yan^1^, Yimin Zhang^1^, Ping Zhu^1^, Xianghong Li^2^, Limin Sang^3^, Ming Zhang^3^, Wenhe Huang^4^, Lianxing Lin^5^, Jundong Wu^4^, Yue Xin^3^, Junhui Fu^5^, Li Zhang^3^, Shuming Zhang^1^, and *Jiang Gu^1,6^

^1^ Provincial Key Laboratory of Molecular Pathology, Department of Pathology and Pathophysiology, Shantou University Medical College, Shantou, 515041, Guangdong, China

^2^ Department of Pathology, Key Laboratory of Carcinogenesis and Translational Research, Beijing University Cancer Hospital and Institute, Beijing, 100142, China

^3^Dalian Municipal Central Hospital, Dalian, 116033, Liaoning, China

^4^ Cancer Hospital of Shantou University Medical College, Shantou, 515041, Guangdong, China

^5^ Shantou Central Hospital, Shantou, 515041, Guangdong, China

^6^ Jinxin Research Institute for Reproductive Medicine and Genetics, Chengdu Xi Nan Gynecological Hospital, Sichuan, Chengdu, China

**Supplementary figure legends:**

**Fig. S1**. Tumor tissue/cells grew faster in ACM. **a.** Calculations of implant and growth areas. The area of a freshly implanted tumor (left; 40x) was calculated based on the mean diameter (measured in three different directions of the tissue image), which was then divided by two to get the radius (r). The total area $(\boldsymbol{A}\boldsymbol{1}\text{)}=\pi r^{2}$ was finally divided by the amplification factor (40x). The tumor growth area calculation is shown by the schematic diagram (right), the blue area represents implanted tissue (*A^1^*) and the white area represents the increase in tumor area (*A^2^ – A^1^*). The formula for the calculation is provided below. **b.** Size comparison of new growths in different culture conditions. Six solid tumors (breast cancers) were cultured with ACM and FBS methods side-by-side. The size of new growths was measured at days 5, 8, and 10. *: Student T-test, p≤0.05. **c.** Images of liquid cancer cultures, taken on Days 2 and 4. Many more cells survived in ACM by Day-2, and tissue-like structures formed in ACM by Day 4, but not in FBS culture. LSC (lung squamous cell carcinoma); LAC (lung adenocarcinoma). **d.** Multiple cell types in implanted samples. H&E stain for 4% formalin fixed fresh tumor samples. Left: A solid tumor of gastric adenocarcinoma that used for implantation in 3D-culture, wherein the cancer nest, infiltrated lymphocytes and matrix tissue are designated with orange, blue and green boundaries respectively. Right: Cells isolated from ascites of a patient with gastric adenocarcinoma. Cancer cells, mesothelial cells and lymphocyte identified by pathologist are circled with orange, green, and blue respectively.

**Fig. S2.** Differences in growth patterns between ACM and FBS. **a**. Serial images of solid tumor (LAC) in cultures and the H&E stain by day-24. **b.** Serial images of a BDC (pleural effusion sample) in cultures and the Live/Dead fluorescence stain by day-7 (cells were ≥ 80% confluence). Well-organized structures formed in ACM but not FBS, as observed in both solid and body fluid samples.

**Fig. S3.** Difference between ACM and ECM cultures of. **a.** Solid tumor. A breast adenocarcinoma was side-by-side cultured in ACM and two ECM (serum from two other patients with breast adenocarcinoma) individually for 15 days. In ACM, the new growth became much wider and thicker, relative to in ECM. IT: implanted tumor tissue. **b.** Pleural effusion sample. Cells isolated from a pleural effusion sample of lung cancer were side-by-side cultured in ACM and in three ECM (pleural effusions from other lung cancer patients) individually for 48 hours. Cell viability was measured with Live/Dead cell image kit and Nuclear Dyes-Live (ThermoFisher Scientific; Waltham, MA USA). Green: live cells; red: dead cells; blue: nuclear, and yellow: stack effect of three colors.

**Fig. S4. Immunophenotype similarity between parental and ACM cultured gastric cancer**. Cells isolated from an ascites sample were cultured with ACM and then harvested on day-7. The expressions of CK (cytokeratin), CEA (carcinoembryonic antigen), and CR (Calretinin) in new growths were compared to freshly isolated cells (scale bar = 60 μm).

**Fig. S5. EGF and TGF-β levels in serum.** ELISA analyses for EGF and TGF-β production in culture media of ACM and FBS for breast cancer (solid tumor samples; n=6). The ACM and FBS were collected on the same day of culture for each case (usually after 10 – 15 days). The average concentration for ACM is shown in red column and for FBS in black. The concentration of each culture well for a tumor was indicated by square dots (ACM) and by triangle (FBS). In FBS medium, there were no detectable EGF and very low TGF-β before and after culture. In ACM, EGF and TGF-β remained in media both prior to and after culturing. Student ***t***-test: * p<0.05, ** p<0.01, *** p<0.001, and **** p<0.0001.

**Fig. S6**. 3D-ACM culture processes. **a**. for solid tumors. **b**. for malignant body fluids (detailed procedures are described in the Methods section).

**Supplementary video legends:**

**Supplementary video 1:** Self-organization of solid tumor (Gastric Cancer) in ACM culture. Using the recording function of the microscope (AE2000, Motic, China), the tissue was photographed every 10 min, for 48 hours. Recording began on Day-10 of the culture. Cells migrated from IT (implanted tissue) and expanded in a radiated pattern, in a well of a 24-well plate.

**Supplementary video 2:** Self-organization of liquid tumor (Breast Ductal Carcinoma) in ACM culture. Using the recording function of the microscope AE2000, the tissue was photographed every 30 min, for 48 hours. Recording began on Day-2 of the culture. Cells organized themselves and formed a tissue-like structure in a culture dish with a 100 mm diameter.
